# Size-dependent magnetic tuning of lateral photovoltaic effect in nonmagnetic Si-based Schottky junctions

**DOI:** 10.1038/srep46377

**Published:** 2017-04-11

**Authors:** Peiqi Zhou, Zhikai Gan, Xu Huang, Chunlian Mei, Yuxing Xia, Hui Wang

**Affiliations:** 1 State Key Laboratory of Advanced Optical Communication Systems and Networks, School of Physics and Astronomy, and Key Laboratory of Thin Film and microfabrication of the Ministry of Education, Shanghai JiaoTong University, 800 Dongchuan Rd, Shanghai 200240, P. R. China

## Abstract

In this article, we report a magnetic tuning lateral photovoltaic effect (LPE) in a nonmagnetic Si-based Schottky junctions. In the magnetic field intensity range of 0 to 1.6 T, the variation amplitude of LPE sensitivity is as high as 94.8%, the change of LPV is and the change rate of lateral photo-voltage even reaches 520 mV/T at 1.5 T, which is apparently higher than the results of previous reported researches in magnetic materials. This effect is attributed to the combined result of the influence of magnetic field on diffusion current and the rectification property of our anisotropic structure. This work may expand the application of LPE in magnetism field such as magnetic sensor and magnetoresistance, and it suggests a new way to investigate the carrier transport in Schottky junctions under magnetic field.

The modification of the charge transport process may induce magnetic effect in conventional nonmagnetic semiconductors, which has been the focus of intensive study in recent years. As examples, unevenly distributed carriers in Si have been reported to be sensitive to magnetic field^1^,^2^,^3^. In these cases, an external current source is essential to maintain the asymmetric distribution of electrons. Compared to electric injection, optical injection should be another advisable alternative if the photo currents can distribute themselves unevenly for some reason in transport process. Guided by this strategy, we recently found such a magnetic tuning lateral photovoltaic effect (LPE) in a nonmagnetic metal-semiconductor structure. LPE is a light involved physical effect, and its most important feature is the lateral photo voltage (LPV) varies linearly with irradiation position with high sensitivity. Since its discovery in 1930^4^, a large number of studies have been conducted to explain this effect^5^,^6^,^7^,^8^,^9^ and improve the sensitivity and linearity of LPE in varieties of PN junction type or MOS type structures, such as interface modification^10^, external bias^11^,^12^, quantum dots embedding^13^, ions implantation^14^, and so on^15^,^16^. However, magnetic field controlled LPE was only reported in magnetic materials with quite small variation^17^,^18^. In this paper, we realized large-range control of LPE by magnetic field with sensitivity as high as 520 mV/T. We attribute this result to the asymmetric diffusion of photo currents in well-designed structure. This work suggests an approach to investigate the carrier transport in Schottky junctions, and it may expand the application space of LPE in magnetic sensor and control.

## Methods

**Figure 1:**
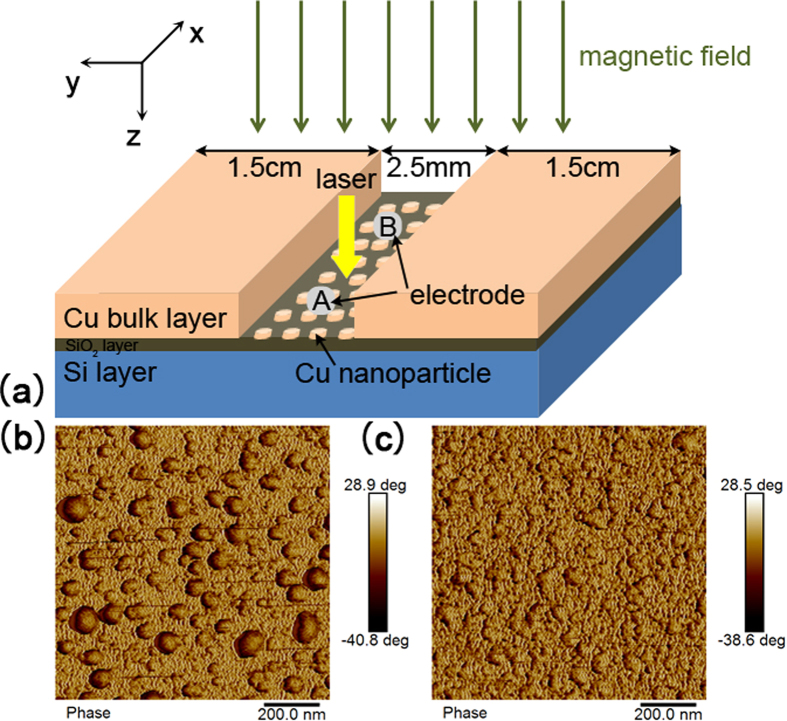
(**a**) Schematic illustration of the sample structure. (**b**) AFM images of the slit area, showing discontinuous Cu nanoparticles, and the average thickness of this area is 2.46 nm. (**c**) AFM images of the bulk Cu layer, showing continuous film with an average thickness of 16.4 nm.

Figure 1(a) shows the schematic illustration of our sample, which presents a metal-semiconductor (MS) structure. However, differing from other conventional MS structures, the metal layer in our sample is consisted of two parts: a slit with discontinuous Cu nanoparticles and bulk Cu layers on its both sides. This two parts of Cu films were both deposited on n-type Si (1 1 1) (with a natural oxide layer about 1.2 nm) at room temperature by radio-frequency sputtering. The base pressure of the vacuum system prior to deposition was 4.1 × 10 − 4 Pa, and the argon gas pressure was 0.7 Pa during the deposition. The deposition rates, determined by stylus profile meter on thick calibration samples, were 0.41 Å/s. Two complementary masks were used to deposit the two Cu parts separately. Firstly, a narrow mask (2.5 mm width) was covered on the silicon surface to deposit the bulk Cu layer for 400 s (16.4 nm thickness), and then the other mask was covered on the complementary area to deposit the slit for 60 s (2.46 nm thickness). The AFM images (see Supplementary Figs S1 and S2 for more images) of sample surface are shown in Fig. 1(b,c), which indicates that the slit region films are discontinuous nanoparticles and the other region are bulk Cu layer in light of Volmer-Weber growth mode^19^,^20^. To measure the lateral photovoltage, two electrodes (less than 1 mm in diameter, see A and B in Fig. 1(a)) contacting to the slit were formed by alloying indium and showed no measurable rectifying behavior. A semiconductor laser (635 nm wavelength) was used to scan the sample, with a 100 μm laser spot diameter and a 5 mW laser power reaching the sample.

## Results and Discussion

**Figure 2:**
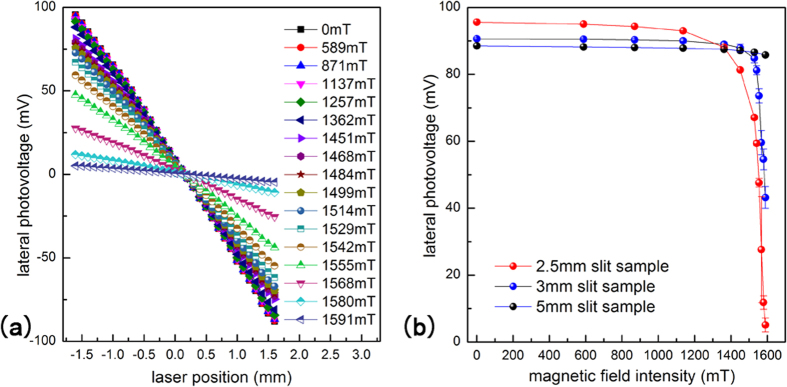
(**a**) The evolution of LPV curves under different magnetic field intensities. (**b**) When laser is fixed on a certain point, the LPV of different slit width samples change as a function of magnetic field intensity.

Figure 2(a) Shows the evolution of LPV curves under different magnetic field intensities. We can see that the sensitivity of LPV (the slope of the curve) reduces with the increase of magnetic field intensity. The original sensitivity (without magnetic field) of LPV is 57.4 mV/mm, and it drops to 3.0 mV/mm when magnetic field intensity reaches 1.59 T. The decreasing amplitude is as high as 94.8%. This is a very interesting result, because we have never found a more than 3% variation in uniform metal film structures, whether the thickness is 2.46 nm or 14.6 nm. In other words, such an obvious change of LPE was only observed in this “slit structure”. To further investigate this phenomenon, we fixed the laser point (close to point A) and studied the response of LPV in different silt width structures, which is shown in Fig. 2(b). For the red curve (corresponding to 2.5 mm slit sample), we can see two magnetic field intensity zones with obviously different slopes. Before 1.3 T, the decrease of LPV is very slow, only 5.4 mV/T, so this zone can be called “nonsensitive zone”. However, when the magnetic field intensity increases from 1.3 T to 1.6 T, the drop of LPV become much more pronounced, and the slope reaches 520 mV/T, which is nearly 100 times greater than the nonsensitive zone. So we call this zone “sensitive zone”. Comparing to the cases of other samples, we found that the boundary of the two zones is closely related to the width of the slit. For the 3 mm slit sample (shown in blue line), the curve inflection point is about 0.2 T behind the red line’s inflection point. And for the 5 mm slit sample (shown in black line), we can’t even find the “sensitive zone” in our limited magnetic field intensity range.

**Figure 3:**
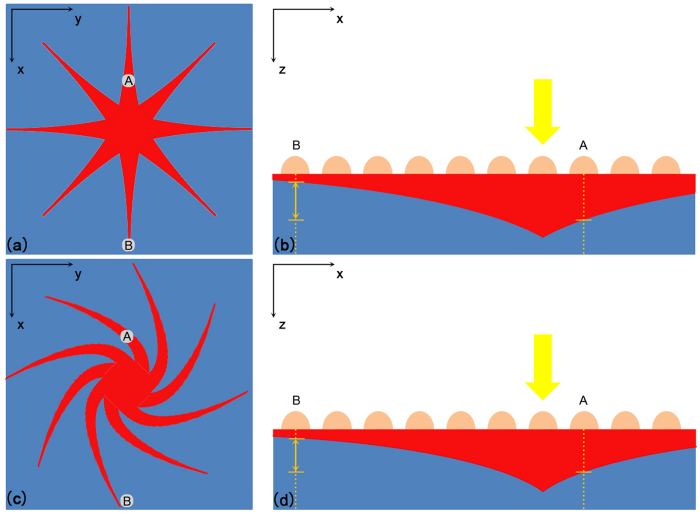
(**a**) The original diffusion currents (formed by photo-generated holes) distribution of a uniform Cu (without slit) -Si structure without magnetic field. (**b**) The distribution of generated holes intensity along the line of electrodes A and B without magnetic field. (**c**) The diffusion currents distribution of a uniform Cu-Si structure under a high intensity magnetic field. (**d**) The distribution of generated holes intensity along the line of electrodes A and B under a high intensity magnetic field.

Given the nonmagnetic property of our structure, the influence of magnetic field on electric current is the only possible explanation for our experimental phenomena. Figure 3 Shows the formation of LPV in a uniform Cu (2.46 nm average thickness)-Si structure (the oxide layer is not shown in the figure because of its tunneling thickness). When a laser (the photon energy is greater than the energy gap of Si) is irradiating at a certain point on the surface, photon-generated carriers will be continuously injected and diffuse in all directions. For n-type Si film, electrons are majority carriers and have a much higher migration rate than holes. As a reasonable approximation, the photon-generated electrons instantaneously redistribute themselves uniformly^9^ over the Si layer, while the photon-generated holes are gradiently distributed from the laser point to around. In this case, the value of LPV mainly depends on the distribution of generated holes, which is shown in Fig. 3b. Assuming that the holes density of each point is D_A_ and D_B_, thereby the LPV is positive correlation with 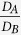
21,22 in a qualitative analysis (see Supplementary Fig. S3). The diffusion currents formed by photon-generated holes are shown as red branches in Fig. 3a, and the reducing width of each branch presents the decrease of current caused by recombination during diffusion. When a magnetic field along Z-axis is applied on the sample, if Cu film is uniform and isotropic, the diffusion currents in all directions will deflect a same angle, just as shown in Fig. 3c. For point A, although the original diffusion current deviates, there will be currents in other directions turning to this area, so the holes density D_A_ changes quite little. This is why we can’t find any more than 3% variation of LPV in uniform metal film structures.

**Figure 4:**
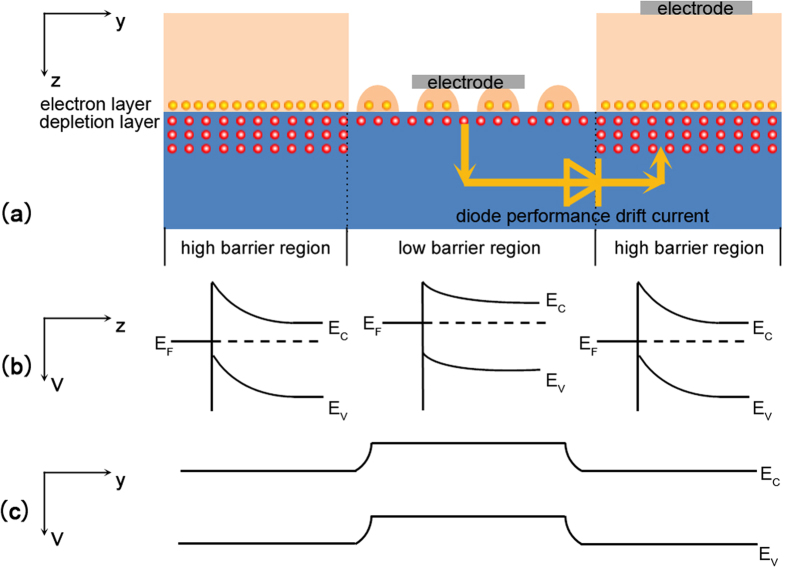
(**a**) The schematic of the structure as three side by side Schottky junctions. (**b**) The energy band diagrams of the junctions. (**c**) The distribution of the conduction band and valence band of Si layer in the direction of y axis.

**Figure 5:**
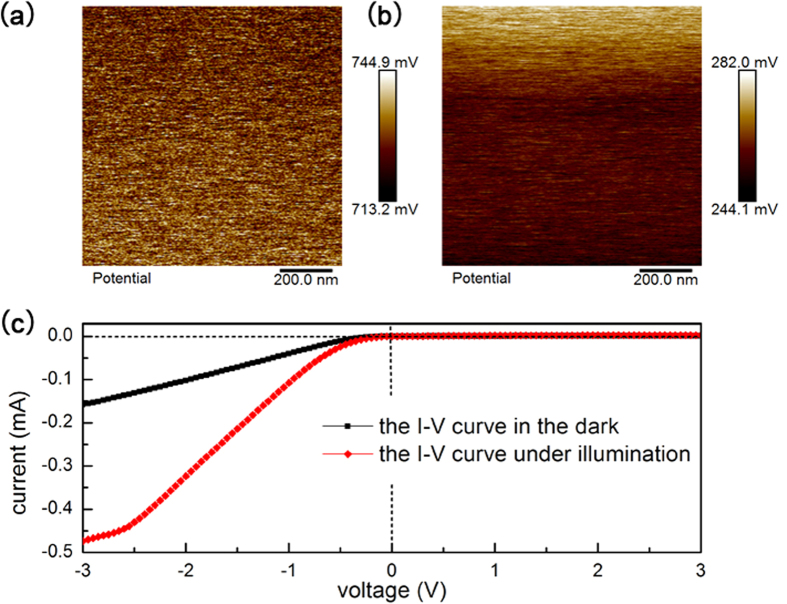
(**a**,**b**) The surface potential scan images of the slit area and bulk layer area with Kelvin Probe Force Microscopy (KPFM), which can be seen as an evidence of the difference of the three Schottky barriers. (**c**) The I-V characteristics between the two electrodes shown in Fig. 4(a), showing diode performance of drift current between different barrier height junctions.

**Figure 6:**
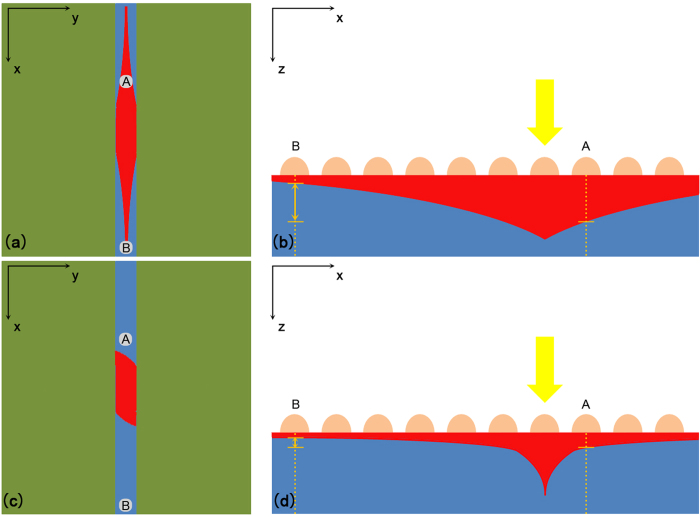
(**a**) The original diffusion currents distribution of anisotropic Cu (with slit)-Si structure without magnetic field. (**b**) The distribution of generated holes intensity along the line of electrodes A and B without magnetic field. (**c**) The diffusion currents distribution of a uniform Cu-Si structure under a high intensity magnetic field. (**d**) The distribution of generated holes intensity along the line of electrodes A and B under a high intensity magnetic field.

However, this compensation of diffusion current will be broken when the Cu film is anisotropic. Just as our structure, the Cu film is formed by a slit with discontinuous Cu nanoparticles and bulk Cu layers on its both sides, which can be seen as three side by side Schottky junctions. Although the material of metal layer is the same Cu, the Schottky barrier height of the middle junction is much lower than the other two junctions’ due to the low coverage of Cu nanoparticles^23^. As shown in Fig. 4a, in the middle slit area, the depletion layer is thinner than the other areas’, which means a weaker built-in electric field or in other words, a lower Schottky barrier height. The energy band diagrams of the junctions are shown in Fig. 4b and c in different views, from which we can see energy bands of different junctions are in different levels. At the edge of the slit, the energy bands bend downwards and form a lateral built-in field which is similar to a PN junction. The existence of this lateral field will restrict the diffusion of the excited holes from the slit toward its two sides, and prevent electrons diffusing from the two sides into the slit in the meantime. So the difference of energy band will result in a unilateral conductivity in the y direction. The difference of Schottky barrier height is indirectly verified by the surface potential scan with Kelvin Probe Force Microscopy (KPFM) shown in Fig. 5a,b, from which we can see the Fermi levels of the middle junction and the other junctions are different. Also the rectification property has already been verified by the I–V characteristics shown in Fig. 5c. (The electrodes of the I–V characteristics measurement were shown in I–V Fig. 4a. Although the electrodes are contacted to the metal layer, the current can only take a detour through the Si due to the extremely low conductivity^24^,^25^ of the discontinuous Cu nanoparticles just as shown in Fig. 4a.) In Fig. 5c, the black line is the I–V curve in the dark, which shows a typical unidirectional continuity. As to the red line, the I–V curve under illumination, the unidirectional continuity didn’t change, which means that the lateral barrier shown in Fig. 4c is high enough to guarantee that ambipolar diffusion. If not, the forward current in the red line will increase markedly due to the increase of carrier concentration. So the I–V curves shown in Fig. 5c can prove the unilateral conductivity of carriers between different junctions in Si layer. Therefore, when a laser is irradiating on the surface, the photon-generated holes can only diffuse in the x direction, just as shown in Fig. 6a. When this current deflects in magnetic field, there will be no other currents turning to this area to compensate the drop of holes density, as a consequence, D_A_ will significantly decrease and so will the LPV, which is shown in Fig. 6b.

**Figure 7:**
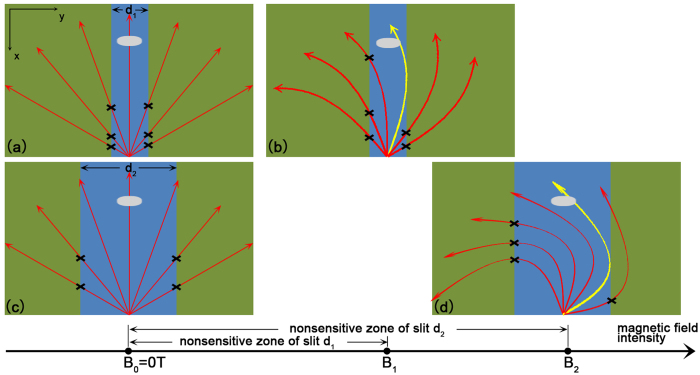
A schematic of the size-dependent property of this magnetic tuning lateral photovoltaic effect. (**a**) The diffusion currents distribution of narrow slit (width d_1_) sample without magnetic field. (**b**) The diffusion currents distribution of narrow slit sample under the critical magnetic field intensity, the current on the far right of the low barrier region (yellow branch) can exactly pass through the right end of the electrode. (**c**) The diffusion currents distribution of wide slit (width d_2_) sample without magnetic field. (**d**) The diffusion currents distribution of wide slit sample under the critical magnetic field intensity.

The size-dependent character of this magnetic tuning lateral photovoltaic effect can be qualitatively explained in Fig. 7. The red branches represent the diffusion current formed by photo-generated holes, and they will gradually deflect more and more observably with the increase of magnetic field intensity. For a certain slit width d_1_, there is a critical magnetic field intensity B_1_ as shown in Fig. 7b. Under this intensity, the current on the far right of the low barrier region (yellow branch shown in the figure) can exactly pass through the right end of the electrode. Before the magnetic field intensity reaches B_1_, the diffusion currents can always completely cover the electrode, so the holes density collected by electrode changes little and so does the LPV. However, after the magnetic field intensity reaches B_1_, the yellow branch will scan from right to left on the electrode with the increase of magnetic field intensity. Given that there is no current on the right of the yellow branch in low barrier region, the holes density collected by electrode will obviously reduce with the increase of magnetic field intensity, so B_1_ is actually the boundary of nonsensitive zone and sensitive zone. Just as shown in Fig. 7(c),(d), the critical magnetic field intensity will naturally increase from B_1_ to B_2_ when the width of slit increases from d_1_ to d_2_. So there is a remarkable correlation between the silt width and the width of nonsensitive zone, which indicates that reducing the silt width is an effective way to move the sensitive zone to lower magnetic field intensity range. This model also suggests that the sensitive zone will not be quite long, although it’s not shown in our data due to the limit magnetic field intensity range of our equipment, there will be another nonsensitive zone after the yellow branch turns to the left side of the electrode.

In conclusion, we have discovered a size-dependent magnetic tuning of lateral photovoltaic effect (LPE) in nonmagnetic Schottky junctions. This work indicates that LPE can be quite susceptible to magnetic field in some well-designed structures, even though the material itself is nonmagnetic. We contribute this phenomenon to the influence of magnetic field on the asymmetrical distributed diffusion current in semiconductor layer. This work suggests a new way to investigate and control the carrier transport in Schottky junctions, and it may expand the application of LPE in magnetic magnetism field.

## Additional Information

**How to cite this article**: Zhou, P. *et al*. Size-dependent magnetic tuning of lateral photovoltaic effect in nonmagnetic Si-based Schottky junctions. *Sci. Rep.* **7**, 46377; doi: 10.1038/srep46377 (2017).

**Publisher's note:** Springer Nature remains neutral with regard to jurisdictional claims in published maps and institutional affiliations.

## Supplementary Material

Supplementary Information
