# Supplementary material for: Size-dependent magnetic tuning of lateral photovoltaic effect in nonmagnetic Si-based Schottky junctions
Source: Sci Rep. 2017 Apr 11;7:46377. doi: 10.1038/srep46377 (PMC5387727; doi:10.1038/srep46377)
Supplement: Supplementary Information [file srep46377-s1.pdf]

Size-dependent magnetic tuning of lateral photovoltaic effect in nonmagnetic Si-based Schottky junctions

Supplementary information

Peiqi Zhou, Zhikai Gan, Xu Huang, Chunlian Mei, Yuxing Xia and Hui Wang*

State Key Laboratory of Advanced Optical Communication Systems and Networks, School of Physics and Astronomy, and Key Laboratory of Thin Film and microfabrication of the Ministry of Education, Shanghai JiaoTong University, 800 Dongchuan Rd, Shanghai 200240, P. R. China

*__Corresponding author:__

Email: huiwang@sjtu.edu.cn

PRAT 1: The evidence that Cu film is discontinuous nano-particles.

Figure S1: The height image, phase image and 3D image of the 2.46 thickness sample, showing that Cu films are actually discontinuos.

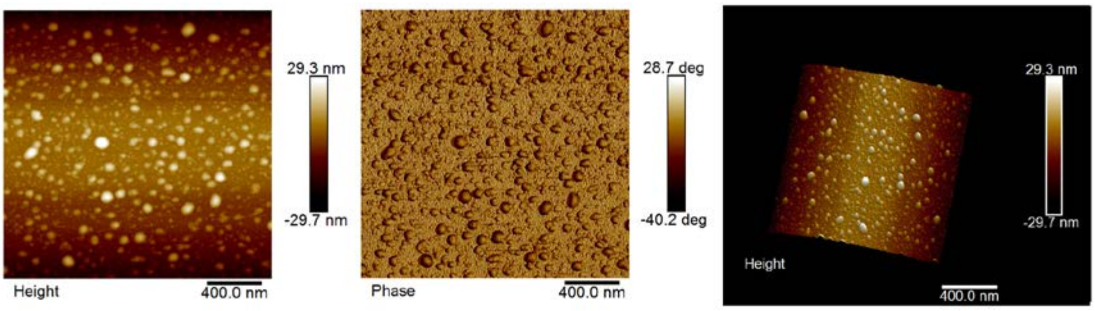

Figure S2: The AFM images of different samples' surfaces in increasing order of the metal film average thickness， showing a typical Volmer-Weber growth mode.

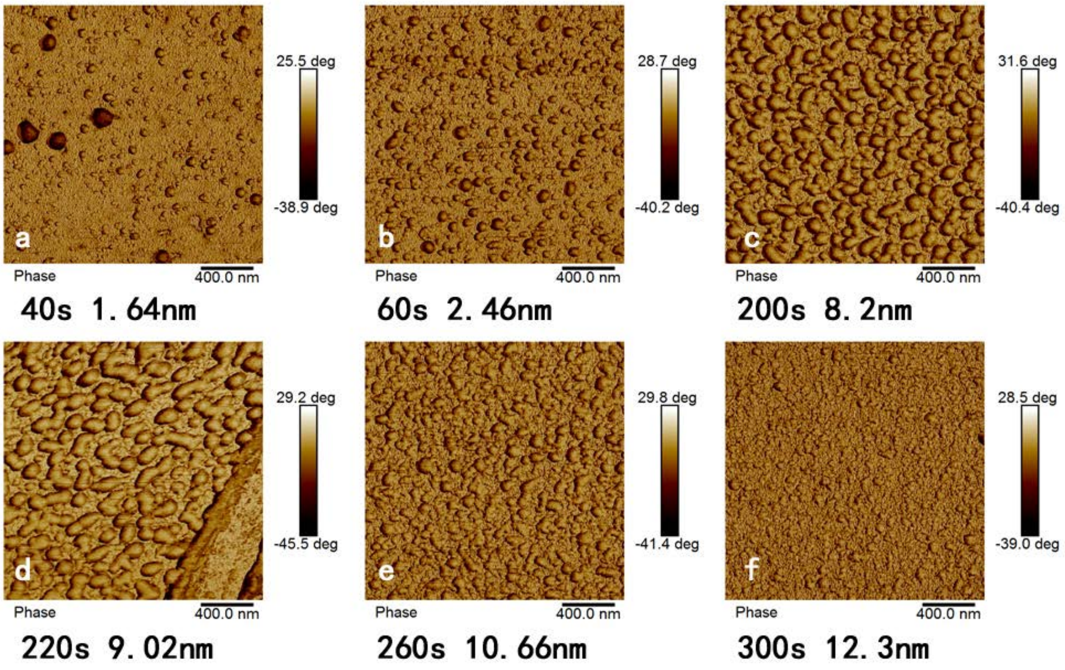

PART 2: The derivation of LPV in theory.

There are two ways to present the value of LPV. One is using the theory of Hirohiko Niu[1], who modified Lucovsky's theory[2] in some respects (Martins' work is established on Lucovsky's theory, which is not suitable for our work because the metal layer in our experiment is not equipotential), considering that the metal layer is quasi-continuous(the Cu particles are still seen as very thin film), and the derivation is as follow[3]:

$$\frac{d^2 J}{dx^2} = \alpha^2 J \tag{1}$$

$$\alpha^2 = \frac{qJ_s}{kT}\left(\frac{\rho}{t} + \frac{\rho'}{t'}\right) \tag{2}$$

$$J_s = A^* T^2 \exp\left(-\Phi / kT\right) \tag{3}$$

$$V(x) = \left(\frac{dqf_x\rho}{t\alpha}\right)\frac{\cosh\alpha\left(l - x_L\right)}{\sinh\alpha l}\left(\cosh\alpha l - \cosh\alpha x_L\right) \tag{4}$$

$$V(0) - V(l) = \left(\frac{2dqf_x\rho}{t\alpha}\right)\frac{\sinh\left(\alpha l / 2\right)}{\sinh\alpha l}\sinh\alpha\left(\frac{l}{2} - x_L\right) \tag{5}$$

When αl <1, $LPV = \left(\frac{2dqf_x\rho}{t\alpha}\right)\left(\frac{1}{2} - \frac{x_L}{l}\right)$ (6)

where α is the spatial decay (fall-off) parameter, *Js* is the Schottky current, ρ is the metal film resistivity, ρ' is the semiconductor film resistivity, *t* the metal film thickness, *t'* the semiconductor film thickness, *A** is Richardson constant, Φ is Schottky barrier height, *d* is the diameter of the light spot, f_x is the light generated carrier flux in x direction, *l* is the distance between two electrodes, and x_L is the position of the light spot.

When a high-intensity magnetic field is applied on our sample, all these parameters remain unchanged except f_x (the film resistivity may increases slightly due to geometric magnetoresistance effect, but this change is negligible comparing with the change of f_x). It is because that the original current in x direction deflects in magnetic field, and there are no other currents turning to this area. Therefore, f_x decreases rapidly with the increase of magnetic field intensity, and so does the LPV.

Another explanation doesn't need the quasi-continuous assumption of the Cu film: just as shown in Figure S3, the diffusion of holes will enhance the intensity of built-in field at each point, as a result, the electrons in Cu particles will move into Si layer and lead to a drop of the quasi-Fermi level of the electrons in Cu particles. If we assume that the number of electrons injected into Si layer is proportional to the holes density (which is a reasonable approximation), then LPV can be expressed by the difference of quasi-Fermi levels between each point in x direction. The derivation is as follow[4,5]:

$$N_p\left(x\right) = N_p\left(0\right)\exp\left(-\frac{x}{\lambda}\right) \quad \text{(diffusion equation in the semiconductor)} \tag{1}$$

$$\lambda=\sqrt{D\tau}\ , \tag{2}$$

$$\Delta n \propto \Delta p\ , \tag{3}$$

$$E_{Fn} = E_F + k_0 T \ln(\Delta n / n) \tag{4}$$

$$LPV = \{[\mathrm{E}_{Fn}(A) - \mathrm{E}_{Fn}(B)]/(-q)\} = (kT/q)\ln[\Delta n(A)/\Delta n(B)] \propto \Delta p(A)/\Delta p(B) \tag{5}$$

Both these two explanations are agreeable to our experimental results and we finally choose the second one in our paper because this explanation is more visual for readers unfamiliar with LPE.

Figure S3: The schematic diagram of the second explanation.

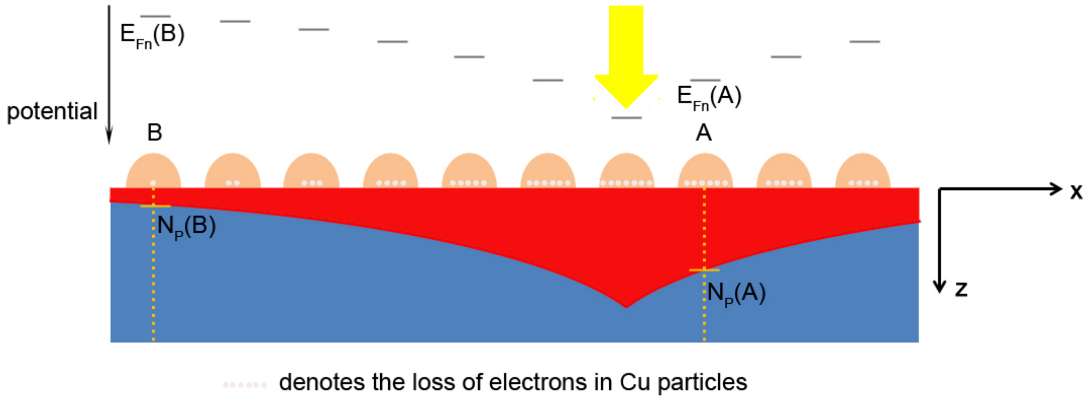

References

1. Niu, H., Matsuda, T., Sadamatsu, H. & Takai, M. Application of Lateral Photovoltaic Effect to the Measurement of the Physical Quantities of P-N Junctions-Sheet Resistivity and Junction Conductance of N_2^+ Implanted Si. *Jpn. J. Appl. Phys.* **15,** 601–609 (1976).
2. Lucovsky, G. Photoeffects in Nonuniformly Irradiated p-n Junctions. *J. Appl. Phys.* **31,** 1088 (1960).
3. Levine, B. F., Willens, R. H., Bethea, C. G. & Brasen, D. Wavelength dependence of the lateral photovoltage in amorphous superlattice films of Si and Ti. *Appl. Phys. Lett.* **49,** 1608 (1986).
4. Yu, C. Q., Wang, H., Xiao, S. Q. & Xia, Y. X. Direct observation of lateral photovoltaic effect in nano-metal-films. *Opt. Express* **17,** 21712–21722 (2009).
5. Lu, J. & Wang, H. Large lateral photovoltaic effect observed in nano Al-doped ZnO films. *Opt. Express* **19,** 13806–13811 (2011).
